# Plasma Nitrate Levels Are Related to Metabolic Syndrome and Are Not Altered by Treatment with DPP-4 Inhibitor Linagliptin: A Randomised, Placebo-Controlled Trial in Patients with Early Type 2 Diabetes Mellitus

**DOI:** 10.3390/antiox10101548

**Published:** 2021-09-29

**Authors:** Melanie Reijrink, Stefanie A. De Boer, Anniek M. Van Roon, Riemer H. J. A. Slart, Bernadette O. Fernandez, Martin Feelisch, Hiddo J. L. Heerspink, Harry Van Goor, Jan-Luuk Hillebrands, Douwe J. Mulder

**Affiliations:** 1 Medical Center Groningen, Department of Internal Medicine, Division of Vascular Medicine, University of Groningen, 9713 Groningen, The Netherlands; m.reijrink@umcg.nl (M.R.); s.a.de.boer@umcg.nl (S.A.D.B.); a.m.van.roon01@umcg.nl (A.M.V.R.); 2 Medical Center Groningen, Department of Nuclear Medicine and Molecular Imaging, University of Groningen, 9713 Groningen, The Netherlands; r.h.j.a.slart@umcg.nl; 3 Department of Biomedical Photoacustic Imaging (BMPI), University of Twente, 7522 Enschede, The Netherlands; 4 Faculty of Medicine, Clinical and Experimental Sciences, University of Southampton, SO17 1BJ Southamptonc, UK; B.Fernandez@soton.ac.uk (B.O.F.); M.Feelisch@soton.ac.uk (M.F.); 5 Medical Center Groningen, Department of Clinical Pharmacy and Pharmacology, University of Groningen, 9713 Groningen, The Netherlands; h.j.lambers.heerspink@umcg.nl; 6 Medical Center Groningen, Department of Pathology and Medical Biology, Division of Pathology, University of Groningen, 9713 Groningen, The Netherlands; h.van.goor@umcg.nl (H.V.G.); j.l.hillebrands@umcg.nl (J.-L.H.)

**Keywords:** diabetes, inflammation, linagliptin, metabolic syndrome, nitrate, nitric oxide, oxidative stress, vegetable intake

## Abstract

The depletion of nitrate and nitrite, stable nitric oxide (NO) end-products, promotes adipose tissue dysfunction and insulin resistance (IR). Dipeptidyl peptidase-4 (DPP-4) inhibitors have the potentially beneficial side effect of increasing NO availability. In this study, nitrate and nitrite levels and the effects of DPP-4 inhibitor linagliptin were investigated in relation to metabolic syndrome (MetS) markers. Treatment-naive patients with early type 2 diabetes mellitus (T2DM) (*n* = 40, median age 63 IQR (55–67) years, 63% male, mean HbA1c 45 ± 4.4 mmol/mol) were randomized (1:1) to linagliptin (5 mg/day) or placebo. MetS-related markers (body mass index (BMI), triglycerides, HOMA-IR, gamma-glutamyltransferase (GGT), C-reactive protein (CRP), and adiponectin), plasma levels of nitrate, nitrite, total free thiols (TFT) and vegetable intake were estimated at baseline and after 4 and 26 weeks of treatment. Plasma nitrate, but not nitrite, correlated positively with vegetable intake (r = 0.38, *p* = 0.018) and was inversely associated with HOMA-IR (r = −0.44, *p* = 0.006), BMI (r = −0.35, *p* = 0.028), GGT (r = −0.37, *p* = 0.019) and CRP (r = −0.34, *p* = 0.034). The relationship between nitrate and HOMA-IR remained significant after adjusting for BMI, CRP, vegetable intake and GGT. With stable vegetable intake, nitrate and nitrite, TFT, adipokines and CRP did not change after 26 weeks of linagliptin treatment. While plasma nitrate is inversely associated with MetS, linagliptin treatment does not significantly influence nitrate and nitrite concentrations, oxidative stress, adipose tissue function and systemic inflammation.

## 1. Introduction

Type 2 diabetes mellitus (T2DM) is a worldwide health problem and it is often associated with metabolic syndrome (MetS) [1,2]. According to the World Health Organisation, MetS is a pathologic condition characterized by abdominal obesity, insulin resistance (IR), hypertension, and hyperlipidaemia [3]. Patients with T2DM often present clear MetS characteristics in early, noninsulin dependent disease stages. Obesity and IR have been shown to produce a state of oxidative stress and low-grade inflammation [4,5], leading to adipose and vascular tissue dysfunction, ultimately resulting in cardiovascular complications [5]. Based on the above, interventions aiming at reducing cardiovascular risk by decreasing oxidative stress and inflammation should preferably start in patients with incipient T2DM. Systemic oxidative stress is strongly associated with increased cardiovascular risk in patients with T2DM [6,7,8], although specific underlying mechanisms remain unclear and require further study.

Since oxidative stress decreases the availability of bioactive nitric oxide (NO) due to scavenging, the NO pathway could be an important mediator in this enhanced risk. The consecutive production of the pro-oxidant, peroxynitrite leads to the uncoupling of endothelial NO synthase, which becomes a dysfunctional superoxide-generating enzyme [9,10]. On the other hand, NO and its stable end-products, nitrate and nitrite, have been shown to exert antioxidant, anti-obesity and anti-diabetic effects in nonhuman studies [11,12]. However, there is conflicting data about the protective effects of nitrate and nitrite on oxidative stress in patients with T2DM [13]. In most human studies describing the association between nitrate and nitrite with markers of the MetS, the dietary contribution to circulating nitrate/nitrite levels was not taken into consideration [14,15,16]. Dietary nitrate is metabolized to nitrite by the oral microbial flora and may produce NO under certain conditions, and nitrate intake can be elevated with increased consumption of green leafy vegetables and beetroots [17,18]. For instance, the intake of spinach improves NO status, lipid homeostasis and decreases inflammation in mice [19]. However, in clinical practice, changes in diet are generally difficult to implement due to compliance-related aspects, stressing the need for drug-oriented interventions.

Dipeptidyl peptidase-4 (DPP-4) inhibitors are glucose-lowering drugs with potentially beneficial additional effects [20]. DPP-4 inhibitors may modulate the NO system and other important markers of MetS, such as adipose tissue function. In experimental and animal studies, DPP-4 inhibitors have been shown to reduce oxidative stress and increase NO availability [21,22,23]. DPP-4 inhibitors may enhance nitrate and nitrite production and improve adipose tissue function by increasing glucagon-like peptide-1 and levels of adiponectin, and by decreasing insulin and leptin levels [23,24,25,26,27]. Importantly, however, these effects of DPP-4 inhibitors have thus far not been demonstrated to occur in humans. We, therefore, sought to confirm that DPP-4 inhibitor treatment increases circulating NO metabolite concentrations, positively affects MetS markers and reduces oxidative stress and inflammation.

We here studied nitrate and nitrite, as stable end-products of NO formation, in relation to markers of MetS in patients with early T2DM. Furthermore, we assessed the effects of linagliptin monotherapy on nitrate and nitrite plasma concentrations, oxidative stress, adipose tissue function and inflammatory markers of MetS, in the same patient cohort.

## 2. Materials and Methods

### 2.1. Aim, Design and Setting of the Study

The primary aim of this study was to investigate the association between nitrate and nitrite concentrations and markers indicative of MetS. Secondly, we aimed to increase nitrate and nitrite levels in patients with early stage T2DM by DPP-4 inhibition, and determine its effects on MetS markers. This study is a subanalysis of the single centre, randomized, double-blind, prospective, placebo-controlled, parallel-group RELEASE study. The RELEASE study was conducted from February 2014 to March 2016 to investigate the effects of 26 weeks DPP-4 inhibitor linagliptin in patients with early T2DM [28].

The study was approved by the Medical Ethical Institutional Review Board of the University Medical Centre Groningen (number 2013-080) and carried out according to the principles of the Declaration of Helsinki and Good Clinical Practice guidelines. All participants gave written informed consent. The trial was registered with clinicaltrials.gov (accessed on 25 August 2021) (NCT02015299).

### 2.2. Patient Characteristics

Study design and population have been described in detail previously [29]. In short, patients were males and females ≥30 and ≤70 years of age, with T2DM according to American Diabetes Association criteria, and receiving a stable dose of blood pressure and/or lipid lowering medication. Exclusion criteria included: use of glucose lowering drugs, previous cardiovascular disease(s) and uncontrolled hypertension (systolic blood pressure (SBP) > 160 mmHg or a diastolic blood pressure (DBP) > 100 mmHg). In the RELEASE study, patients were randomized to oral linagliptin 5 mg once daily or a matching placebo for 26 weeks, stratified for age (30–49 versus 50–70 years), drug use and smoking status. For this substudy, 40 RELEASE study patients were included based on the availability of stored plasma samples.

### 2.3. Dietary and Laboratory Assessments

In addition to biochemical assessments, SBP and food frequency questionnaires were taken at baseline and after 26 weeks. No dietary restrictions were imposed and no dietary advice was given during the study. Intake of energy and nutrients was scored retrospectively, and subsequently analysed and quantified by a dietician. The food consumption of one month was expressed in mean intake of several nutrient groups in grams per day. At baseline and after 4 and 26 weeks, blood samples were obtained in the morning after >8 h of overnight fasting. Glycaemic indices (plasma glucose, insulin, HbA1c), lipid profile, liver function (alanine aminotransferase (ALT), aspartate aminotransferase (AST), and gamma-glutamyl transferase (GGT)) and systemic inflammatory parameters (C-reactive protein (CRP) and leukocyte counts) were measured using routine automated assays. Estimated glomerular filtration rate (eGFR) was calculated as a marker of kidney function using the CKD-EPI formula [30]. Liver function parameters ALT, AST, and GGT were measured as an indication of non-alcoholic fatty liver disease (NAFLD). IR was estimated with the homeostasis model assessment using fasting insulin and glucose concentrations (HOMA-IR: fasting insulin * fasting glucose/22.5) [31].

In addition to assessment of standard clinical laboratory parameters, plasma was stored at −80 °C for future analysis. Nitrate and nitrite levels were measured by high-pressure liquid ion chromatography, with on-line reduction of nitrate to nitrite and post-column Griess diazotisation (ENO20 Analyser; Eicom, Kyoto, Japan), as previously described in detail [32,33]. Total free thiol (TFT) concentration, as a marker of systemic oxidative stress, was measured by comparing the absorbance readings of plasma reacted with Ellman’s reagent to that of freshly prepared L-cysteine standards [34,35]. Reduced TFT levels reflect increased systemic oxidative stress. Total plasma adiponectin and leptin plasma levels were determined with an ELISA kit (Linco Research, St. Charles, MO, USA; EZHADP-61K and EZHL-80SK).

### 2.4. Statistical Analyses

Data from all available plasma samples were included in the analysis and missing values were not imputed. Univariate correlations were analysed with Pearson’s or, when not normally distributed, Spearman’s correlation coefficient (R). Based on significant univariate correlations of nitrate with markers of MetS and vegetable intake, multiple linear regressions were performed. Five models were constructed to evaluate the addition of MetS markers on the association of nitrate with HOMA-IR and to assess whether HOMA-IR was associated with nitrate independently of anticipated markers. The interaction term HOMA-IR * BMI was added because of a strong correlation between these factors. After including HOMA-IR, BMI, and the interaction term (model 2), CRP (model 3), vegetable intake (model 4), and GGT (model 5) were added. Between-group differences from baseline to 4 and 26 weeks were analysed using the calculated deltas (Δ). An ANCOVA model was constructed with change from baseline (i.e., Δ nitrate and nitrite plasma levels, vegetable intake or MetS markers) as a dependent variable, randomization group as fixed factor and baseline levels as covariate. All analyses were performed with SPSS (2013, IBM SPSS Statistics, Version 22.0. Armonk, NY, USA: IBM Corp). *p*-value of <0.05 was considered statistically significant.

## 3. Results

In total, 40 patients with T2DM were included in this study. The baseline characteristics of these 40 patients are presented in Table 1. The median age was 63 years, 63% of the participants were male, mean HbA1c was 45 mmol/mol and mean diabetes duration was 1 year. The baseline characteristics were well balanced between the linagliptin and placebo group.

### 3.1. Stable End-Products of NO in Relation to MetS Markers

**Figure 1:**
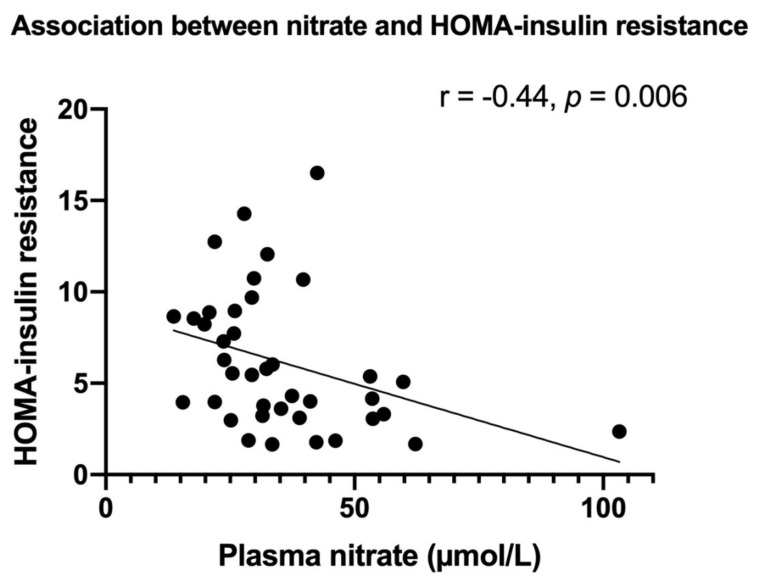
Nitrate plasma level was inversely related to HOMA-insulin resistance. Correlation between plasma nitrate concentration (as measured by high-performance liquid chromatography and expressed in µmol/L) and HOMA-insulin resistance (HOMA-IR, fasting insulin * fasting glucose/22.5).

**Figure 2:**
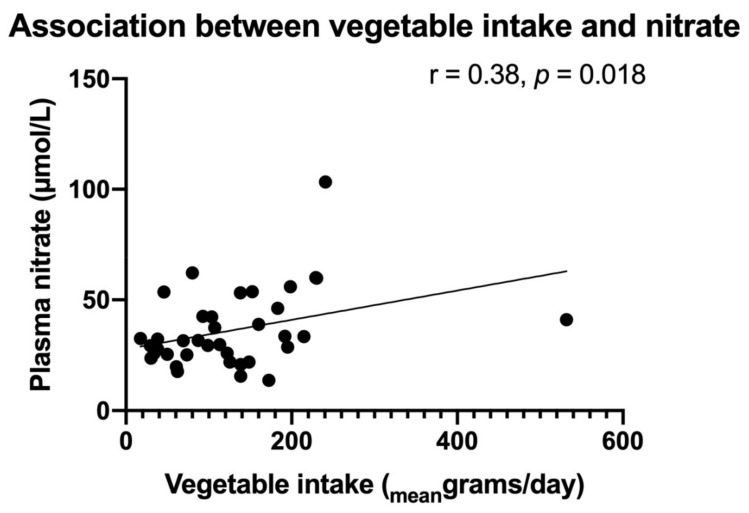
Food frequency questionnaire assessed vegetable intake was associated with plasma nitrate level. Correlation between mean daily vegetable intake (in mean grams/day) and plasma nitrate concentration (as measured by high-performance liquid chromatography and expressed in µmol/L).

At baseline, fasting nitrate concentrations were inversely related to HOMA-IR (r = −0.44, *p* = 0.006) (see Figure 1), BMI (r = −0.35, *p* = 0.028), GGT (r = −0.37, *p* = 0.019), and to inflammatory markers CRP (r = −0.34, *p* = 0.034) and leukocyte count (r = −0.47, *p* = 0.002). Furthermore, nitrate was borderline significantly inversely related to triglycerides (r = −0.30, *p* = 0.056) and a similar trend was observed for ALT (r = −0.29, *p* = 0.067). Nitrate was not associated with HbA1c, TFT, eGFR, AST, HDL-, LDL- and total cholesterol, nor blood pressure (data not shown). Vegetable intake correlated positively with nitrate (r = 0.38, *p* = 0.018, Figure 2), inversely with HOMA-IR (r = −0.45, *p* = 0.005) and CRP (r = −0.61, *p* < 0.001) and showed an inverse association, with borderline significance versus leukocyte counts (r = −0.31, *p* = 0.055). Adiponectin was positively associated with vegetable intake (r = 0.38, *p* = 0.018) and showed a positive trend in relation to nitrate (r = 0.27, *p* = 0.074). Leptin showed an inverse relation to vegetable intake (r = −0.34, *p* = 0.037), but was not associated with nitrate (r = −0.25, *p* = 0.11).

The association between nitrate and HOMA-IR was modified by BMI (interaction HOMA-IR * BMI (*p* = 0.013)), with a stronger association in non-obese individuals, and this interaction variable was included in the multiple linear regression models. Furthermore, adding CRP, vegetable intake, and GGT separately did not alter the association between nitrate and HOMA-IR (Table 2).

Unlike nitrate, nitrite did not show any significant associations with MetS including HOMA-IR, BMI, GGT, CRP, leukocyte count, triglycerides or ALT, but correlated inversely with vegetable intake (r = −0.34, *p* = 0.037) and showed a trend with TFT (r = 0.31, *p* = 0.055).

### 3.2. Effects of the DPP-4 Inhibitor Linagliptin

#### 3.2.1. Effects of Linagliptin Treatment on Nitrate and Nitrite Plasma Concentrations

At baseline, nitrate was 33 (27–48) μmol/L and nitrite was 0.14 (0.092–0.35) μmol/L in the linagliptin group and, respectively, 29 (25–43) μmol/L and 0.20 (0.0094–0.33) μmol/L in the placebo group. Nitrate and nitrite levels did not change significantly after 26 weeks treatment with linagliptin (nitrate and nitrite levels were 38 (29–56) μmol/L and 0.12 (0.11–0.14) μmol/L compared to 49 (30–56) μmol/L and 0.12 (0.11–0.15) μmol/L after 26 weeks treatment with placebo). According to the difference in nitrate and nitrite in change from baseline between linagliptin vs. placebo was 3.18 vs. 15.2 Δμmol/L (*p* = 0.168) and −0.0842 vs. −0.1031 Δμmol/L (*p* = 0.901), respectively. Between baseline and 26 weeks, the amount of vegetable intake did neither change in the overall cohort (110 mean grams/day at baseline vs. 114 mean grams/day at 26 weeks (*p* = 0.27)), nor differ between the change over 26 weeks in linagliptin and placebo group (−14.5 Δmean grams/day in linagliptin vs. −11.5 Δmean grams/day in placebo (*p* = 0.247)).

#### 3.2.2. Linagliptin Effects on MetS Markers

The 26-week treatment with linagliptin caused a reduction in HbA1c and triglycerides of −3.33 Δmmol/mol (vs. placebo 0.95 Δmmol/mol (*p* < 0.001)) and −0.20 Δmmol/L (vs. placebo 0.29 Δmmol/L (*p* = 0.019), respectively. The HOMA-IR was decreased after 26 weeks linagliptin treatment, but did not differ significantly in linagliptin vs. placebo (−1.32 vs. 0.83 (*p* = 0.096)). No significant effects of linagliptin vs. placebo were observed for TFT (−37.7 vs. −37.2 ΔµM (*p* = 0.70)), adiponectin (0.73 vs. 0.18 Δng/mL (*p* = 0.51)), leptin (−0.58 vs. −0.66 Δng/mL (*p* = 0.99)), CRP (−0.055 vs. 0.479 Δmg/l (*p* = 0.25)) and leukocyte counts (−0.05 vs. −0.12 Δ*109/L (*p* = 0.63)). Changes in BMI, liver function and kidney function (GGT, ALT, AST, eGFR), lipids and SBP did not differ after 26 weeks of treatment between linagliptin and placebo.

## 4. Discussion

The major finding of our study is the inverse relationship between plasma nitrate levels and HOMA-IR, BMI, GGT and systemic inflammation and its association with vegetable intake. As expected, linagliptin treatment had a favourable effect on HbA1c and triglycerides; unexpectedly, however, it did not significantly change either nitrite, nitrate, TFT, adipokines or systemic inflammatory parameters. These data show that circulating nitrate levels, but not nitrite, are closely related to MetS and that linagliptin treatment does not significantly affect the concentration of NO metabolites, oxidative stress, adipose tissue function and systemic inflammation in patients with early T2DM.

In this placebo-controlled randomized control trial we found that nitrate alone was related to MetS markers in patients with incipient T2DM. Several experimental studies previously assessed the importance of NO metabolites in diabetes mellitus, showing anti-obesity and anti-diabetic properties [11,12,18,19]. Due to the analytical challenges to accurately quantify the low concentrations of nitrite in complex biological fluids, NO production was often assessed by measurement of the sum of nitrate and nitrite. However, while both products are generated during oxidative NO metabolism, each has its own distinct biological activity. For this reason, we opted to quantify nitrate and nitrite concentrations discretely. Our analyses suggest that nitrate and nitrite concentrations mark different processes in vivo. An increased intake of nitrate is associated with lower lipid accumulation and inflammation and promotes insulin secretion, glucose uptake, mitochondrial efficiency and browning of adipose tissue [7,15,18,36,37,38]. Therefore, increased nitrate concentrations could play an important role in attenuating MetS and T2DM. While the literature is inconsistent about the relation of nitrate and nitrite with IR, previous studies showed that HOMA-IR was associated with nitrate in patients with T2DM and in obese children [14,39]. However, these studies did not correct for diet, and vegetable intake is the main source of nitrate. In the current study, we confirmed its correlation with plasma nitrate and we did correct for nitrate intake in our analyses. Moreover, we noticed that nitrate correlated inversely with GGT-assessed liver function and systemic inflammatory markers. In line with these results, nitrate also showed a borderline-significant inverse correlation with triglycerides and ALT-assessed liver function. These data suggest that increasing plasma nitrate levels might result in favourable effects on MetS.

Obesity, T2DM and oxidative stress contribute to lower NO bioavailability due to decreased NO production and accelerated NO removal, as a result of the uncoupling of the NO synthase, NO scavenging with formation of peroxynitrite, and TNF-α/inflammation and hypoxia-associated alterations in NO metabolism [12]. Furthermore, decreased NO availability causes dysfunction of vascular and adipose tissue, which stimulates the development of IR [7,38]. This in turn promotes inflammation and oxidative stress. Nevertheless, previous studies have demonstrated that dietary nitrate and nitrite administration was associated with increased nitrate and nitrite levels in adipose tissue in obesity and T2DM [12,40,41]. Our study underlines the importance of nitrate in MetS and showed that exogenous (i.e., dietary) nitrite/nitrate intake does not significantly affect the relationship between nitrate and MetS markers.

Furthermore, our data suggest that plasma nitrate might positively affect adiponectin production, as a marker of adipose tissue function. Adipose tissue dysfunction is an important cornerstone of MetS and T2DM by driving metabolic dysfunction, and is characterized by adipocyte hypertrophy, altered adipokine production (i.e., adiponectin and leptin) and lipid metabolism, and local inflammation [42,43,44]. In patients with MetS and T2DM, decreased adiponectin and increased leptin levels were related to increased oxidative stress and systemic inflammation and decreased insulin sensitivity [45,46]. The borderline-significant association between plasma nitrate and adiponectin levels has not been demonstrated before. However, the effects of nitrate and nitrite in relation to adipose tissue function were studied previously. The intake of nitrate and nitrite was found to be related to adiponectin level in visceral adipose tissue and adiponectin plasma concentration was related to nitrite levels [47,48]. Since adiponectin has protective effects on systemic inflammation and IR, the positive trend between nitrate and adiponectin might be of interest for patients with MetS and T2DM.

The protective properties of DPP-4 inhibitors in relation to oxidative and inflammatory parameters in MetS could be of significance for patients with T2DM, in particular in relation to vascular and adipose tissue dysfunction. Recently, linagliptin has shown favourable effects on HbA1c, fasting plasma glucose, triglycerides and arterial inflammation and vascular repair [49,50]. However, in contrast to our expectation, neither nitrate, nitrite, adipokines, TFT nor inflammatory markers were significantly altered after 26 weeks of linagliptin treatment. While animal studies have demonstrated the anti-inflammatory and antioxidant effects of DPP-4 inhibitors, these favourable effects have not been confirmed in humans. Consistent with the findings of earlier studies, the present investigation did not show major effects of DPP-4 inhibitors on oxidative stress and inflammatory status in patients with T2DM [51,52]. However, 12 weeks of linagliptin treatment decreased malondialdehyde-modified LDL, a marker of oxidative stress, but not systemic inflammation [53]. In addition, the effects on oxidative stress and systemic inflammation could be explained by glycaemic changes, which were significantly decreased in the study of Makino et al. [54]. While DPP-4 inhibitors potentially have beneficial effects on oxidative and inflammatory markers, these effects were not yet confirmed in humans.

A strength of this double blind, placebo controlled randomized control trial in patients with early-phase T2DM is that next to biochemical and biomarker assessments, food frequency questionnaires were taken. In order to study the DPP-4-induced effects on nitrate and nitrite levels without the confounding effects of diet, dietary intake should be monitored and kept stable throughout the study period, as in our current study. However, our study is not without its limitations. First, the duration of treatment in our study was 26 weeks, and it remains unknown whether treatments over several years would change NO metabolites, TFT, adipokines and systemic inflammatory parameters. Second, the results were obtained in relatively healthy people with early T2DM, and although intervention in an early phase of the disease is important, caution is warranted by extrapolating our findings to patients with T2DM with a longer diabetes duration or with advanced metabolic disease. Third, this study was performed in a cross-sectional study design and our data do not inform about causal links between NO metabolites, diet and MetS markers.

## 5. Conclusions

In conclusion, in the current study we demonstrated an association between plasma nitrate concentrations and MetS in treatment-naïve patients with T2DM, independent of dietary intake as a major exogenous source. Although this suggests the involvement of nitrate in MetS, further studies need to investigate if nitrate might be an important endpoint in preventing patients with T2DM for cardiovascular complications and NAFLD. Furthermore, linagliptin does not affect the presence of NO metabolites, nitrate and nitrite, oxidative stress, adipose tissue function and systemic inflammation indicating that this DPP-4 inhibitor has no beneficial oxidative or inflammatory effects. Overall, these observations strengthen our knowledge about these NO metabolites in relation to the MetS and underline that, although vegetable intake is an important source of nitrate, it does not influence the association of nitrate and IR. Larger, prospective studies with nitrate supplementation therapy and non-pharmacological interventions including alterations in nutrition and/or physical activity are needed to substantiate these results and investigate causal links between nitrate and MetS.

## Figures and Tables

**Table 1:** Clinical characteristics of the study population at baseline.

	All (*n* = 40)	Linagliptin (*n* = 21)	Placebo (*n* = 19)
Male (n)	25 (63%)	13 (62%)	12 (63%)
Age (years)	63 (55–67)	63 (52–66)	63 (57–69)
Diabetes duration (years)	1.0 (0.0–3.8)	1 (0–5)	1 (0–3)
Body mass index (kg/m^2^)	30 (27–37)	32 (28–39)	29 (27–34)
Glucose (mmol/L)	7.5 ± 0.92 *	7.6 ± 0.90	7.3 ± 0.95 ^
Insulin (mU/L)	16 (9.8–25)	13 (9.6–27)	18 (9.7–22)
HOMA-insulin resistance	5.4 (3.2–8.7) *	4.3 (3.2–9.3)	5.5 (3.7–8.0) ^
HbA1c (mmol/mol)	45 ± 4.4	45 ± 4.3	45 ± 4.6
C-reactive protein (mg/L)	1.3 (0.73–3.1)	1.6 (0.80–2.9)	1.2 (0.70–3.9)
Leukocyte counts (*109/L)	7.0 ± 1.8	7.2 ± 1.8	6.7 ± 1.8
Total free thiols (µM)	426 (399–441)	411 (374–438)	430 (413–453)
Total cholesterol (mmol/L)	4.7 ± 0.97	4.8 ± 1.2	4.6 ± 0.71
High density lipids (mmol/L)	1.4 ± 0.33	1.3 ± 0.27	1.4 ± 0.39
Low density lipids (mmol/L)	3.1 ± 1.0	3.2 ± 1.2	2.9 ± 0.86
Triglycerides (mmol/L)	1.4 (0.90–2.0)	1.4 (1.1–2.0)	1.2 (0.82–2.1)
Estimated glomerular filtration rate (mL/min*1.73m^2^)	85 (79–97)	91 (81–98)	81 (66–87)
Alanine aminotransferase (U/L)	26 (43–20)	25 (19–41)	27 (22–43)
Aspartate aminotransferase (U/L)	26 (22–33)	24 (21–28)	26 (22–33)
Gamma-glutamyltransferase (U/L)	35 (25–47)	33 (22–39)	36 (27–64)
Nitrate (µmol/L)	32 (25–42)	33 (27–48)	29 (25–43)
Nitrite (µmol/L)	0.19 (0.095–0.33)	0.14 (0.092–0.35)	0.20 (0.094–0.33)
Vegetable intake (mean grams/day)	110 (58–175) °	107 (61–153) ^®^	122 (50–192)
Adiponectin (ng/mL)	8.3 (6.3–10.5)	7.3 (5.7–9.9)	8.5 (6.4–12.6)
Leptin (ng/mL)	13 (6.6–25)	13 (4.6–26)	13 (8.1–23)
Systolic blood pressure (mmHg)	139 ± 13	139 ± 15	138 ± 12

Values are *n* (percentage of the group), mean ± standard deviation or median [and interquartile range]. * *n* = 39, ° *n* = 38, ^ *n* = 18, ^®^ *n* = 19.

**Table 2:** Nitrate and HOMA-IR: multivariate linear regression models.

	Model 1 (R^2^ = 0.191, *p* = 0.005)		Model 2 (R^2^ = 0.327, *p* = 0.003)		Model 3 (R^2^ = 0.345, *p* = 0.005)		Model 4 (R^2^ = 0.354, *p* = 0.014)		Model 5 (R^2^ = 0.356, *p* = 0.030)	
Dependent: nitrate	St. ß	*p*-value	St. ß	*p*-value	St. ß	*p*-value	St. ß	*p*-value	St. ß	*p*-value
HOMA-IR	−0.44	0.005	−1.08	0.001	−1.01	0.004	−0.91	0.015	−0.88	0.033
BMI			−0.49	0.035	−0.46	0.048	−0.43	0.043	−0.43	0.046
Interaction variable HOMA-IR * BMI			1.03	0.013	1.02	0.014	0.85	0.045	0.82	0.060
CRP					−0.16	0.35	−0.10	0.62	−0.10	0.62
Vegetable intake							0.084	0.65	0.080	0.67
Gamma-GT									−0.038	0.82

Five models were constructed to evaluate the association of nitrate with HOMA-IR with addition of MetS markers, to assess whether HOMA-IR was associated with nitrate independently of anticipated markers. The interaction term HOMA-IR * BMI was added because of a strong correlation between these factors. After including HOMA-IR, BMI, and the interaction term (model 2), CRP (model 3), vegetable intake (model 4), and GGT (model 5) were added.

## Data Availability

Data is contained within the article.

## References

[1] GBD 2016 Disease and Injury Incidence and Prevalence Collaborators (2017). Global, regional, and national incidence, prevalence, and years lived with disability for 328 diseases and injuries for 195 countries, 1990–2016: A systematic analysis for the Global Burden of Disease Study 2016. Lancet.

[2] Punthakee Z., Goldenberg R., Katz P., Diabetes Canada Clinical Practice Guidelines Expert Committee (2018). Definition, Classification and Diagnosis of Diabetes, Prediabetes and Metabolic Syndrome. Can. J. Diabetes.

[3] Alberti K.G., Eckel R.H., Grundy S.M., Zimmet P.Z., Cleeman J.I., Donato K.A., Fruchart J.C., James W.P.T., Loria C.M., Smith S.C. (2009). Harmonizing the metabolic syndrome: A joint interim statement of the International Diabetes Federation Task Force on Epidemiology and Prevention; National Heart, Lung, and Blood Institute; American Heart Association; World Heart Federation; International Atherosclerosis Society; and International Association for the Study of Obesity. Circulation.

[4] Herder C., Schneitler S., Rathmann W., Haastert B., Schneitler H., Winkler H., Bredahl R., Hahnloser E., Martin S. (2007). Low-grade inflammation, obesity, and insulin resistance in adolescents. J. Clin. Endocrinol. Metab..

[5] Rehman K. (2017). Akash MSH Mechanism of Generation of Oxidative Stress and Pathophysiology of Type 2 Diabetes Mellitus: How Are They Interlinked?. J. Cell. Biochem..

[6] Folli F., Corradi D., Fanti P., Davalli A., Paez A., Giaccari A., Perego CMuscogiuri G. (2011). The role of oxidative stress in the pathogenesis of type 2 diabetes mellitus micro-and macrovascular complications: Avenues for a mechanistic-based therapeutic approach. Curr. Diabetes. Rev..

[7] Luc K., Schramm-Luc A., Guzik T.J., Mikolajczyk T.P. (2019). Oxidative stress and inflammatory markers in prediabetes and diabetes. J. Physiol. Pharmacol..

[8] Karam B.S., Chavez-Moreno A., Koh W., Akar J.G., Akar F.G. (2017). Oxidative stress and inflammation as central mediators of atrial fibrillation in obesity and diabetes. Cardiovasc. Diabetol..

[9] Förstermann U., Xia N., Li H. (2017). Roles of Vascular Oxidative Stress and Nitric Oxide in the Pathogenesis of Atherosclerosis. Circ. Res..

[10] Albrecht E.W., Stegeman C.A., Tiebosch A.T., Tegzess A.M., van Goor H. (2002). Expression of inducible and endothelial nitric oxide synthases, formation of peroxynitrite and reactive oxygen species in human chronic renal transplant failure. Am. J. Transplant..

[11] Norouzirad R., González-Muniesa P., Ghasemi A. (2017). Hypoxia in Obesity and Diabetes: Potential Therapeutic Effects of Hyperoxia and Nitrate. Oxid. Med. Cell. Longev..

[12] Ghasemi A., Jeddi S. (2017). Anti-obesity and anti-diabetic effects of nitrate and nitrite. Nitric Oxide.

[13] Assmann T.S., Brondani L.A., Boucas A.P., Rheinheimer J., de Souza B.M., Canani L.H., Bauer A.C., Crispim D. (2016). Nitric oxide levels in patients with diabetes mellitus: A systematic review and meta-analysis. Nitric Oxide.

[14] Deb N., Chatterjee S., Mukhopadhyay M., Majumder B., Bhattacharyya S. (2017). Assessment of serum nitrate-nitrite ratio vis-a-vis insulin sensitivity and resistance in type 2 diabetics in a tertiary hospital in Eastern India. Int. J. Res. Med. Sci..

[15] Marchi-Alves L.M., Carnio E.C. (2009). Is There Any Correlation between Insulin Resistance and Nitrate Plasma Concentration in White Coat Hypertensive Patients?. Cardiol. Res. Pract..

[16] Nácul A.P., Andrade C.D., Schwarz P., de Bittencourt P.I. (2007). Spritzer PM Nitric oxide and fibrinogen in polycystic ovary syndrome: Associations with insulin resistance and obesity. Eur. J. Obstet. Gynecol. Reprod. Biol..

[17] Butler A.R., Feelisch M. (2008). Therapeutic uses of inorganic nitrite and nitrate: From the past to the future. Circulation.

[18] Lundberg J.O., Carlström M., Weitzberg E. (2018). Metabolic Effects of Dietary Nitrate in Health and Disease. Cell Metab..

[19] Li T., Lu X., Sun Y., Yang X. (2016). Effects of spinach nitrate on insulin resistance, endothelial dysfunction markers and inflammation in mice with high-fat and high-fructose consumption. Food. Nutr. Res..

[20] Glorie L., D’Haese P.C., Verhulst A. (2016). Boning up on DPP4, DPP4 substrates, and DPP4-adipokine interactions: Logical reasoning and known facts about bone related effects of DPP4 inhibitors. Bone.

[21] Alam M.A., Chowdhury M.R.H., Jain P., Sagor M.A.T. (2015). Reza HM DPP-4 inhibitor sitagliptin prevents inflammation and oxidative stress of heart and kidney in two kidney and one clip (2K1C) rats. Diabetol. Metab. Syndr..

[22] Solini A., Rossi C., Duranti E., Taddei S., Natali A., Virdis A. (2016). Saxagliptin prevents vascular remodeling and oxidative stress in db/db mice. Role of endothelial nitric oxide synthase uncoupling and cyclooxygenase. Vascul. Pharmacol..

[23] Mason R.P., Jacob R.F., Kubant R., Walter M.F., Bellamine A., Jacoby A., Mizuno Y., Malinski T. (2011). Effect of enhanced glycemic control with saxagliptin on endothelial nitric oxide release and CD40 levels in obese rats. J. Atheroscler. Thromb..

[24] Bergmark B.A., Cannon C.P., White W.B., Jarolim P., Liu Y., Bonaca M.P., Zannad F., Morrow D.A. (2017). Baseline adiponectin concentration and clinical outcomes among patients with diabetes and recent acute coronary syndrome in the EXAMINE trial. Diabetes Obes. Metab..

[25] Ida S., Murata K., Betou K., Kobayashi C., Ishihara Y., Imataka K., Uchida A., Monguchi K., Kaneko R., Fujiwara R. (2016). Effect of trelagliptin on vascular endothelial functions and serum adiponectin level in patients with type 2 diabetes: A preliminary single-arm prospective pilot study. Cardiovasc. Diabetol..

[26] Lamers D., Famulla S., Wronkowitz N., Hartwig S., Lehr S., Ouwens D.M., Eckardt K., Kaufman J.M., Ryden M., Müller S. (2011). Dipeptidyl peptidase 4 is a novel adipokine potentially linking obesity to the metabolic syndrome. Diabetes.

[27] Basu A., Charkoudian N., Schrage W., Rizza R.A., Basu R., Joyner M.J. (2007). Beneficial effects of GLP-1 on endothelial function in humans: Dampening by glyburide but not by glimepiride. Am. J. Physiol. Endocrinol. Metab..

[28] De Boer S.A., Hovinga-de Boer M.C., Heerspink H.J., Lefrandt J.D., van Roon A.M., Lutgers H.L., Glaudemans A.W.J.M., Kamphuisen P.W., Slart R.H.J.A., Mulder D.J. (2016). Arterial Stiffness Is Positively Associated With 18F-fluorodeoxyglucose Positron Emission Tomography-Assessed Subclinical Vascular Inflammation in People With Early Type 2 Diabetes. Diabetes Care.

[29] De Boer S.A., Heerspink H.J., Juárez Orozco L.E., van Roon A.M., Kamphuisen P.W., Smit A.J., Slart R.H., Lefrandt J.D., Mulder D.J. (2017). Effect of linagliptin on pulse wave velocity in early type 2 diabetes: A randomized, double-blind, controlled 26-week trial (RELEASE). Diabetes. Obes. Metab..

[30] Levey A.S., Stevens L.A., Schmid C.H., Zhang Y., Castro A.F., Feldman H.I., Kusek J.W., Eggers P., Van Lente F., Greene T. (2009). A new equation to estimate glomerular filtration rate. Ann. Intern. Med..

[31] Matthews D.R., Hosker J.P., Rudenski A.S., Naylor B.A., Treacher D.F., Turner R.C. (1985). Homeostasis model assessment: Insulin resistance and beta-cell function from fasting plasma glucose and insulin concentrations in man. Diabetologia.

[32] Rassaf T., Bryan N.S., Kelm M., Feelisch M. (2002). Concomitant presence of N-nitroso and S-nitroso proteins in human plasma. Free Radic. Biol. Med..

[33] Umbrello M., Dyson A., Pinto B.B., Fernandez B.O., Simon V., Feelisch M., Singer M. (2014). Short-term hypoxic vasodilation in vivo is mediated by bioactive nitric oxide metabolites, rather than free nitric oxide derived from haemoglobin-mediated nitrite reduction. J. Physiol..

[34] Koning A.M., Meijers W.C., Pasch A., Leuvenink H.G., Frenay A.R.S., Dekker M.M., Feelisch M., de Boer R.A., van Goor H. (2016). Serum free thiols in chronic heart failure. Pharmacol. Res..

[35] Abdulle A.E., Bourgonje A.R., Kieneker L.M., Koning A.M., la Bastide-van Gemert S., Bulthuis M.L., Dijkstra G., Faber K.N., Dullaart R.P., Bakker S.J. (2020). Serum free thiols predict cardiovascular events and all-cause mortality in the general population: A prospective cohort study. BMC. Med..

[36] Lundberg J.O., Weitzberg E. (2008). Gladwin MT The nitrate-nitrite-nitric oxide pathway in physiology and therapeutics. Nat. Rev. Drug. Discov..

[37] Jackson J.K., Patterson A.J., MacDonald-Wicks L.K., Bondonno C.P., Blekkenhorst L.C., Ward N.C., Hodgson J.M., Byles J.E., McEvoy M.A. (2018). Dietary Nitrate and Diet Quality: An Examination of Changing Dietary Intakes within a Representative Sample of Australian Women. Nutrients.

[38] Van den Born J.C., Hammes H.P., Greffrath W., van Goor H., Hillebrands J.L. (2016). DFG GRK International Research Training Group 1874 Diabetic Microvascular Complications (DIAMICOM) Gasotransmitters in Vascular Complications of Diabetes. Diabetes.

[39] Ozgen I.T., Tascilar M.E., Bilir P., Boyraz M., Guncikan M.N., Akay C., Dundaroz R. (2012). Oxidative stress in obese children and its relation with insulin resistance. J. Pediatr. Endocrinol. Metab..

[40] Varzandi T., Abdollahifar M.A., Rohani S.A.H., Piryaei A., Zadeh-Vakili A., Jeddi S., Ghasemi A. (2018). Effect of long-term nitrite administration on browning of white adipose tissue in type 2 diabetic rats: A stereological study. Life Sci..

[41] Ohtake K., Nakano G., Ehara N., Sonoda K., Ito J., Uchida H., Kobayashi J. (2015). Dietary nitrite supplementation improves insulin resistance in type 2 diabetic KKA(y) mice. Nitric Oxide.

[42] Friedman J. (2002). Fat in all the wrong places. Nature.

[43] Schrover I.M., Spiering W., Leiner T., Visseren F.L. (2016). Adipose Tissue Dysfunction: Clinical Relevance and Diagnostic Possibilities. Horm. Metab. Res..

[44] Goossens G.H. (2017). The Metabolic Phenotype in Obesity: Fat Mass, Body Fat Distribution, and Adipose Tissue Function. Obes. Facts..

[45] Fasshauer M., Blüher M. (2015). Adipokines in health and disease. Trends Pharmacol. Sci..

[46] Frühbeck G., Catalán V., Rodríguez A., Ramírez B., Becerril S., Salvador J., Portincasa P., Colina I., Gómez-Ambrosi J. (2017). Involvement of the leptin-adiponectin axis in inflammation and oxidative stress in the metabolic syndrome. Sci. Rep..

[47] Kina-Tanada M., Sakanashi M., Tanimoto A., Kaname T., Matsuzaki T., Noguchi K., Uchida T., Nakasone J., Kozuka C., Ishida M. (2017). Long-term dietary nitrite and nitrate deficiency causes the metabolic syndrome, endothelial dysfunction and cardiovascular death in mice. Diabetologia.

[48] Belo V.A., Souza-Costa D.C., Lacchini R., Sertorio J.T., Lanna C.M., Carmo V.P., Tanus-Santos J.E. (2013). Adiponectin associates positively with nitrite levels in children and adolescents. Int. J. Obes..

[49] De Boer S.A., Heerspink H.J., Lefrandt J.D., Hovinga-de Boer M.C., van Roon A.M., Juárez Orozco L.E., Glaudemans A.W., Kamphuisen P.W., Slart R.H., Mulder D.J. (2017). Effect of Linagliptin on Arterial (18)F-Fluorodeoxyglucose Positron Emission Tomography Uptake: A Randomized Controlled Trial (RELEASE). J. Am. Coll. Cardiol..

[50] De Boer S.A., Reijrink M., Abdulahad W.H., Hoekstra E.S., Slart R.H.J.A., Heerspink H.J.L., Westra J., Mulder D.J. (2020). Angiogenic T cells are decreased in people with type 2 diabetes mellitus and recruited by the dipeptidyl peptidase-4 inhibitor Linagliptin: A subanalysis from a randomized, placebo-controlled trial (RELEASE study). Diabetes Obes. Metab..

[51] Bigagli E., Luceri C., Dicembrini I., Tatti L., Scavone F., Giovannelli L., Mannucci E., Lodovici M. (2020). Effect of Dipeptidyl-Peptidase 4 Inhibitors on Circulating Oxidative Stress Biomarkers in Patients with Type 2 Diabetes Mellitus. Antioxidants.

[52] Koren S., Shemesh-Bar L., Tirosh A., Peleg R.K., Berman S., Hamad R.A., Vinker S., Golik A., Efrati S. (2012). The effect of sitagliptin versus glibenclamide on arterial stiffness, blood pressure, lipids, and inflammation in type 2 diabetes mellitus patients. Diabetes Technol. Ther..

[53] Makino H., Matsuo M., Hishida A., Koezuka R., Tochiya M., Ohata Y., Tamanaha T., Son C., Miyamoto Y., Hosoda K. (2018). Effect of linagliptin on oxidative stress markers in patients with type 2 diabetes: A pilot study. Diabetol. Int..

[54] Rizzo M., Rizvi A.A., Spinas G.A., Rini G.B., Berneis K. (2009). Glucose lowering and anti-atherogenic effects of incretin-based therapies: GLP-1 analogues and DPP-4-inhibitors. Expert Opin. Investig. Drugs.

